# Multiple aseptic abscesses and pulmonary involvement in a child with Behcet’s disease phenotype: a case report

**DOI:** 10.3389/fimmu.2025.1550551

**Published:** 2025-06-09

**Authors:** Yucan Zheng, Guiping Kong, Hongmei Guo, Zhifeng Liu, Kunlong Yan

**Affiliations:** Department of Gastroenterology, Children’s Hospital of Nanjing Medical University, Nanjing, China

**Keywords:** Behcet’s disease phenotype, aseptic abscesses, interstitial lung disease, case report, autoimmune diseases

## Abstract

**Background:**

Behcet’s disease (BD) is a lifelong multi-systemic vasculitis disorder that can affect almost any organ. The frequency of involvement of each organ varies depending on demographic and geographical factors. The commonly affected systems include mucosal, articular, ocular, vascular, neurological, and gastrointestinal. Common gastrointestinal manifestations include mucosal ulcers, hemorrhage, and perforation while other phenotypes are very rare.

**Case presentation:**

We describe the case of a 3-year-old boy suffering from BD phenotype with multiple aseptic abscesses (AAs) and interstitial lung disease. Over the past four years since onset, the boy has presented a series of symptoms, including fever, skin necrosis, multiple ulcers in the intestines, multiple aseptic abscesses in the spleen, interstitial lung disease and an isolated abscess in the gastric wall. Through a regimen involving steroids, mercaptopurine, thalidomide endoscopic drainage of the gastric wall abscess, the child’s condition has been effectively managed and improved.

**Conclusion:**

To our knowledge, this is the first reported case of BD phenotype with a gastric wall abscess treated with endoscopic drainage and steroids. Multiple AAs and interstitial lung disease may be the early signs of a BD phenotype in childhood which can respond effectively to glucocorticoids.

## Introduction

Behcet’s disease (BD) is a rare systemic inflammatory disease classified as vasculitis, which can affect almost any organ and is characterized by unpredictable phases of recurrence and remission (1). It is globally distributed, with a prevalence of 10.3 per 100000 people (2), but is particularly prevalent in Middle East and Far East Asia, including populations along the so-called Silk Road. The highest prevalence in observed in Turkey (370 per 100,000 people), followed by Iran, Japan, northern China and Korea (3).

BD is most commonly observed in the third and the fourth decades of life, with occasional cases reported in childhood, accounting for 5-15.5% of all the cases. It affects boys and girls equally, with a male-to-female ratio ranging from 0.6 to 2.1. In pediatric populations, the mean age at onset of BD varies widely, from 4.9 to 12.3 years old (4, 5). Mucocutaneous, musculoskeletal, vascular, neurological and gastrointestinal manifestations are frequently reported (6). In pediatric BD, clinical presentations are heterogeneous and differ among geographic distributions; gastrointestinal involvement is more common in Asia, while vascular manifestations prevail in Middle East and eastern Mediterranean (1). However, worldwide, multiple abscess and interstitial lung disease (ILD) in BD are rare.

Herein, we report a case of a child with BD phenotype presenting with mucocutaneous ulcers, multiple abscess, and ILD, sharing knowledge and experience in this area.

## Case presentation

**Figure 1:**
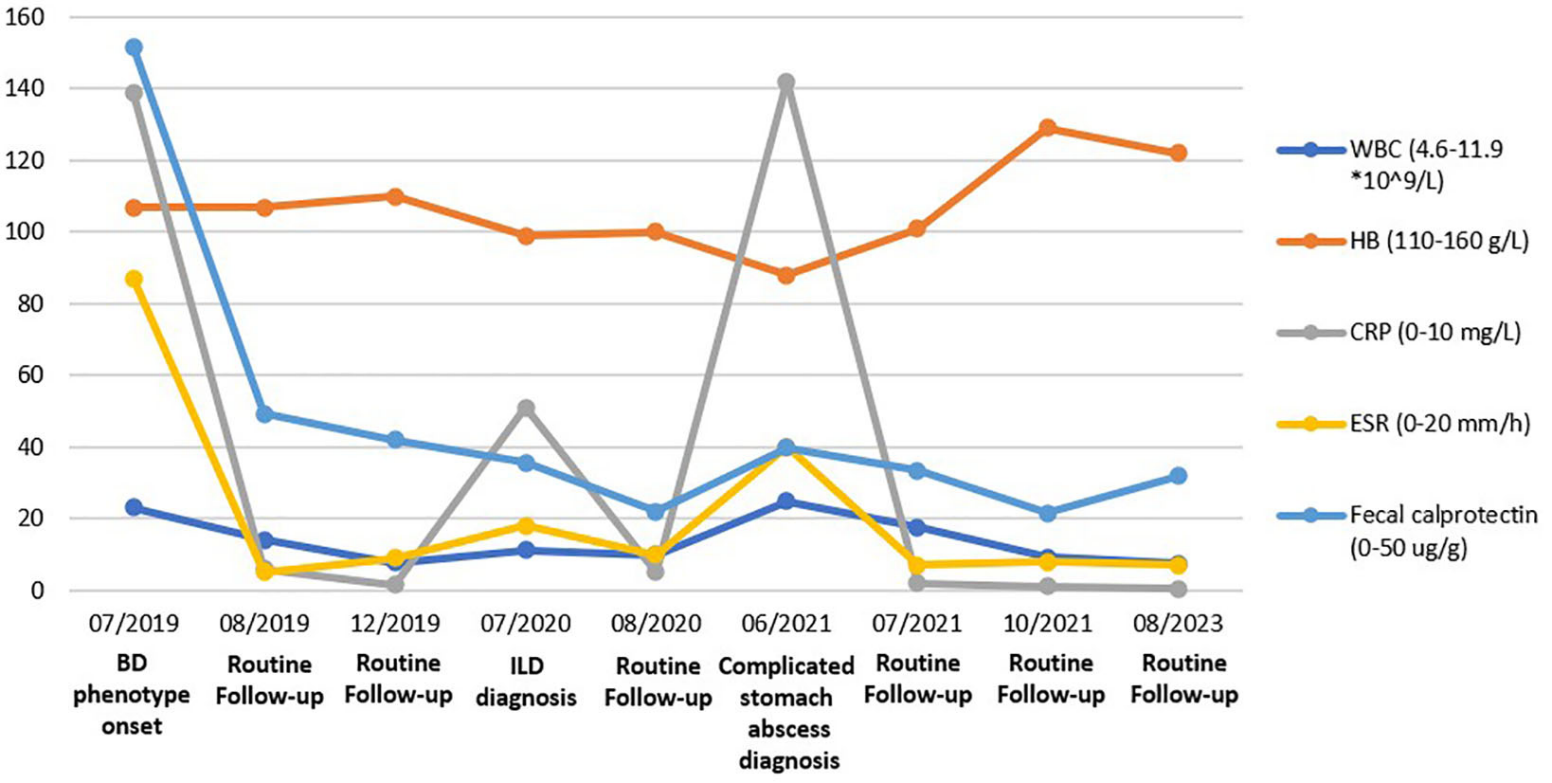
Various labs, including White blood cell counts, Hemoglobin, C-reaction protein, Erythrocyte sedimentation rate, and Fecal calprotectin were trended over four years. The remarkable values for a chosen time frame are included.

**Figure 2:**
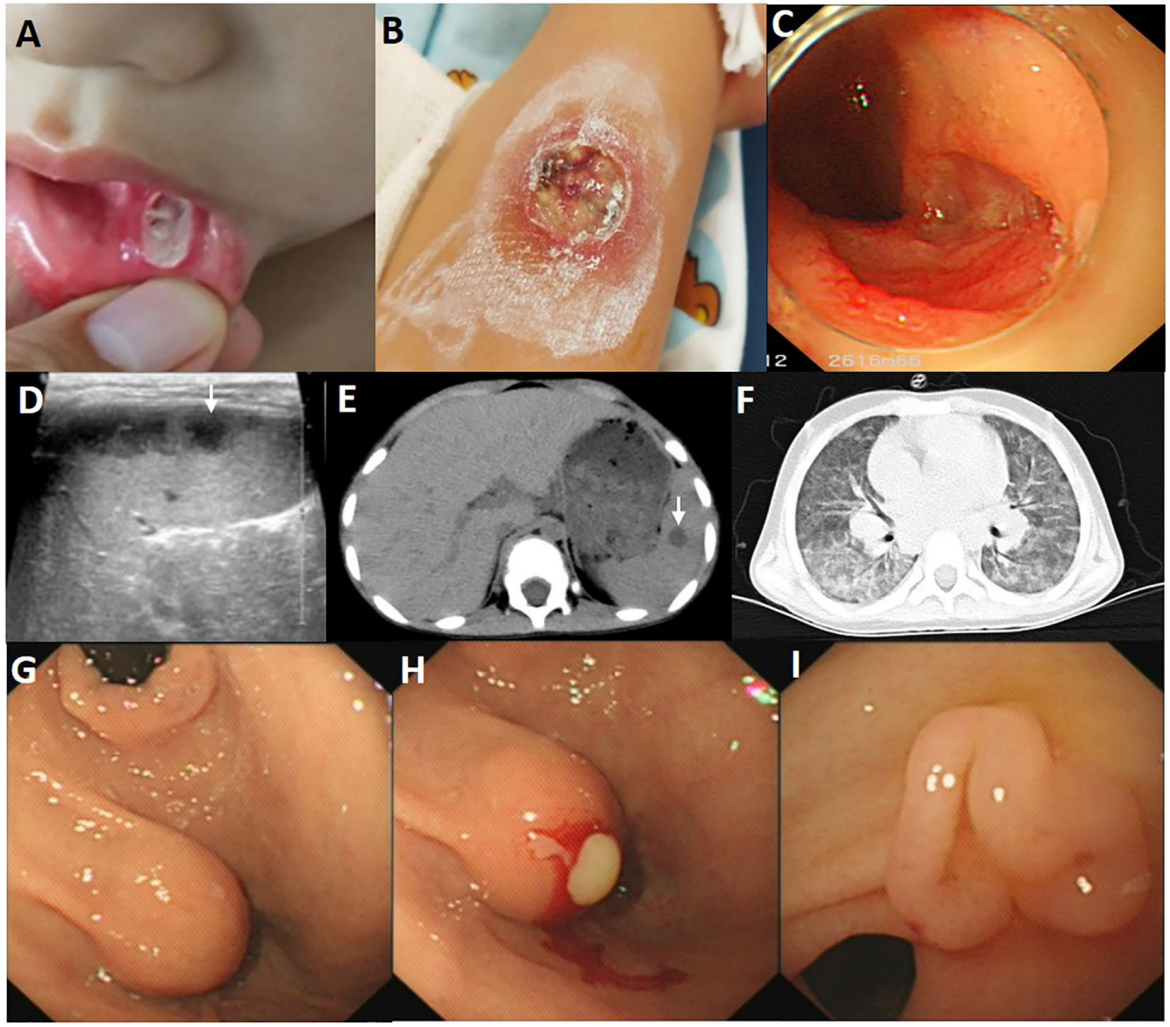
Images of the child with Behcet’s disease phenotype. **(A)** The deep ulcer with a diameter of approximately 8 mm in the buccal mucosa. **(B)** The large ulcer with a diameter of 2cm on the right forearm, with surrounding skin congestion and exudation on the surface. **(C)** A large ulcer with regular edges and smooth bases at the end of ileum. **(D)** Multiple low dark echogenic areas with rough edges on the abdominal ultrasound image, with a maximum of 28*18 mm^2^. **(E)** Multiple circular low-density shadows of varying sizes (a maximum of 14*18 mm^2^) with clear edges in the spleen CT scan at the onset. **(F)** Chest CT images at age of 4.5 years showed diffuse ground glass and reticular opacities in both lung lobes. **(G)** A spherical submucosal protrusion was observed at the small curvature side of gastric antrum, measuring approximately 1.5 cm*1.5 cm. **(H)** Pus was flowing from the submucosal protrusion’s surface. **(I)** The follow-up gastroscopy revealed a cluster of polypoid hyperplasia at the site of the previous abscess at the age of 5 years and 3 months.

**Figure 3:**
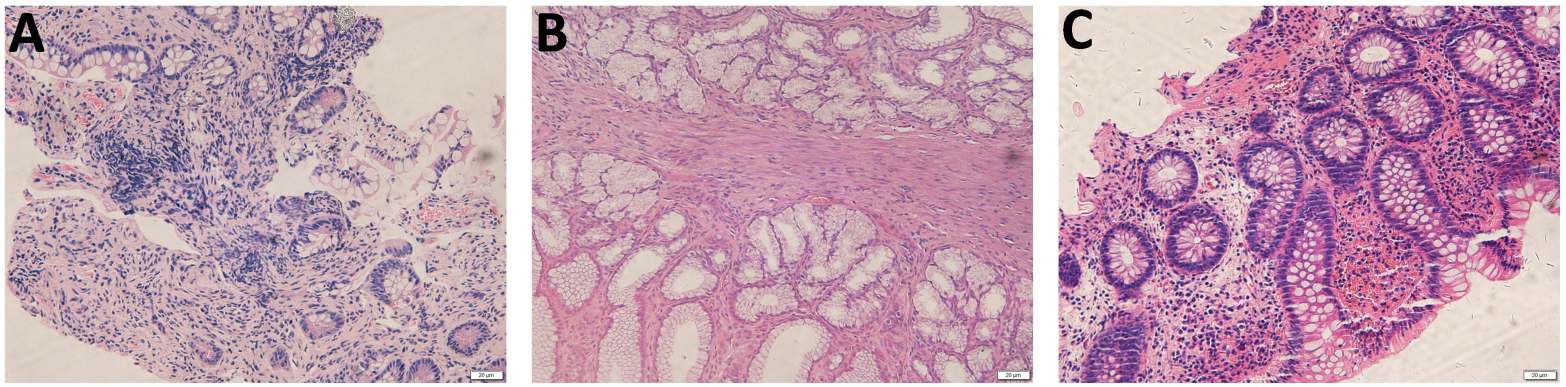
Pathology of the gastrointestinal biopsy. **(A)** Pathology of the terminal ileum at the onset demonstrated remarkable lymphocyte infiltration in the lamina propria. **(B)** Endoscopic pathological biopsy of the gastric antrum performed at the age of 5 years showed a small amount of lymphocyte infiltration in the superficial layer of mucosa. **(C)** Pathology of the ileocecal during disease flare at the age of 7 years demonstrated infiltration of lymphocytes, plasma cells, and eosinophils within the intrinsic membrane.

A 3-year-old boy was an off-spring of two nonconsanguineous healthy parents without any history of any autoimmune or hereditary diseases. He was initially admitted to our hospital with complains of recurrent oral ulcers for two years, intermittent abdominal pain for three months, and a one-kilogram weight loss. Prior to hospitalization, the boy also experienced intermittent fevers for four days and suppurative tonsillitis. Laboratory tests revealed anemia (107 g/L, normal range 110–160 g/L), leukocytosis (23.14*10^9/L, normal range 4.6-11.9*10^9/L), and elevated inflammatory markers, including an erythrocyte sedimentation rate (ESR, normal range 0–20 mm/h) of 87 mm/h and a C-reactive protein (CRP, normal range 0–10 mg/L) level of 139mg/L (**Figure 1**). After receiving a five-day course of amoxicillin and clavulanate potassium at a dosage of 30mg/kg/8h, he gradually developed a remittent fever, a deep ulcer in the buccal mucosa, and a large ulcer on his right forearm (**Figures 2A, B**). Initial investigations included a comprehensive infectious workup, notably negative for bacterial cultures of pharyngeal swabs, blood and urine, as well as negative results for cytomegalovirus, Epstein-Barr virus, human immunodeficiency virus, T-spot, Antistreptolysin O, 1,3-β-D-glucan test and galactomannan antigen test. Despite experiencing mild persistent abdominal pain, fecal calprotectin levels were slightly elevated (151.7 ug/g, normal range 0–50 ug/g). Immunological workup was largely unremarkable, with normal levels of complement components C3 and C4, immunoglobulins IgM, IgG, B cell, T cell and natural killer cells. However, IgA levels were elevated at 2.1g/L, and antinuclear antibody was weakly positive. HLA-B27 was negative. The pathergy test yielded a positive result. Bone marrow examination showed no evidence of malignancy. Abdominal ultrasound revealed a spleen with a regular shape, displaying thickened light spots and multiple low dark echogenic areas with rough edges, including one area measuring up to 28*18 square millimeters which raised suspicion of a splenic abscess confirmed by CT scan (**Figures 2D, E**). Gastroscopy revealed no abnormalities in the upper digestive tract, while colonoscopy showed deep ulcers with regular edges and smooth bases at the end of the ileum and ileocecal valve, with multiple small ulcers were scattered from the ileocecal region to the sigmoid colon (**Figure 2C**). Pathological examination of the intestinal tissue revealed remarkable lymphocyte infiltration in the lamina propria. However, due to the small size of the tissue sample, no signs of vasculitis were observed, only lymphocytic infiltration in the superficial mucosa (**Figure 3**). No eye lesions were found in this patient, including anterior, intermediate, posterior, or panuveitis. There were no clinical signs of neurological involvement such as headache, vomiting, or hemiplegia, and the brain MRI did not reveal any abnormal signals. The patient also exhibited no musculoskeletal involvement, such as myalgia, arthralgia, or restricted joint movement.

Ultimately, according to the guideline (1), the boy was presumptive diagnosed with Behcet’s disease. Therefore, he was promptly initiated on 1.5 mg/kg prednisolone and 2 mg/kg thalidomide. Subsequently, his temperature gradually stabilized, ulcers in the skin and mouth healed, body weigh increased, and inflammation markers decreased to normal levels (**Figure 1**). One month later, the spleen lesions had shrunk to approximately 5*6 mm^2^. Five months later, during a follow-up gastroscopy and splenic ultrasound, gastrointestinal mucosal ulcers were found to have healed, and the hypoechoic area of the spleen had further decreased to 4*3mm^2^.

One year after the initial visit, the boy experienced persistent cough and intermittent shortness of breath for a month. Chest CT imaging revealed increased markings in both lungs, uneven opacity, and diffuse fuzzy patchy shadows throughout (**Figure 2F**). After ruling out multiple pathogenic infections such as bacteria, COVID-19, adenovirus, respiratory syncytial virus, influenza, human metapneumovirus, and mycoplasma through sputum pathogen testing, the child diagnosed with ILD. Treatment included 2 mg/kg of prednisolone, 1.5 mg/kg of mercaptopurine, and 2 mg/kg of thalidomide, in addition to nasal oxygen via a catheter and nebulized budesonide for 20 days. The child’s condition improved, leading to discharge from the hospital. During this hospitalization, whole exome sequencing and karyotype analysis were performed. No pathogenic and structural variants were detected related to Behçet’s disease. Prednisone was gradually tapered off and stopped after two months. Three months later, follow-up chest CT and endoscopy showed significant improvement, with symmetric lung transparency and slightly increased interstitial density. The mucosa of the gastrointestinal tract remained in a relieved state.

Over the subsequent six months, the parents discontinued the medications without medical guidance. When the child reached 5 years of age, irregular fever and oral ulcers recurred, which showed no response to antibiotic treatment. Multiple pathogen tests were repeated, all yielding negative results. However, there was progression of interstitial changes in the lung, characterized by diffuse scattered small nodules in both lungs with ground-glass opacities. CT imaging revealed multiple round low-density shadows within the spleen, with the largest measuring approximately 18mm*16mm. During gastroscopy, a spherical submucosal protrusion was observed at the small curvature side of the gastric antrum, measuring approximately 1.5cm*1.5cm. Pus was observed flowing from the surface, and a large amount of purulent fluid was extracted using an endoscopic injection needle (**Figures 2G, H**). No abnormalities were observed during colonoscopy and pus culture yielded negative results. Consequently, treatment with 2 mg/kg of prednisolone and 2 mg/kg of thalidomide was reintroduced. Three months later, follow-up gastroscopy revealed a cluster of polypoid hyperplasia at the site of the previous abscess on the posterior wall of the junction of the gastric body and fundus (**Figure 2I**).

Over the past year, with maintenance therapy consisting of 2 mg/kg of thalidomide, the boy has experienced steady increases in weight and height, with no recurrence of fever, cough, or ulcers (Various labs are depicted in **Figure 1**). The patient and his parents satisfied with the treatment outcome.

## Discussion

Behcet’s disease exhibits high heterogeneity, and the clinical manifestations across different systems overlap throughout its course, with varying frequencies observed among ethnic group (7, 8). It can affect the skin, mucosa, joints, eyes, vascular system, central nervous system, and gastrointestinal tract (9). The broad spectrum of phenotypes associated with this disease not only distinguishes different patients, but may also characterize different phases of the disease in the same individual (8). Although typically diagnosed in adulthood, the initial symptoms of BD can manifest early and fully developed before the age of 16 years in 4-26% of cases, a condition referred to as pediatric BD (4). Given the absence of validated biomarkers or histological features that unequivocally identify BD, diagnosis primarily relies on clinical signs and symptoms (1, 10). The most commonly used classification criteria for BD were initially developed in 1990 (11) and revised in 2014 (12). In 2016, specific criteria for pediatric BD criteria were established (13). Despite the development of various diagnostic criteria, diagnosing pediatric BD remains challenging, particularly during the initial presentation (5).

The gastrointestinal manifestations of BD share overlapping clinical features with Crohn’s Disease (CD) (14). Both BD and CD can present with abdominal pain, oral ulcers, weight loss, as well as extra-intestinal manifestations such as fever and necrotizing pyoderma that are unresponsive to antibiotics. However, our patient has a characteristic positive Pathergy test for BD and has never exhibited the more typical perianal abscess or fistula findings seen in CD. On endoscopy, this patient’s intestine shows multiple large, deep ulcers that are round or oval, with distinct smooth borders, and appear to be worm-eaten. The ulceration matches the vascular distribution region and lacks the proliferative features and longitudinal distribution characteristic of Crohn’s disease (8). There is also no intestinal stricture. The endoscopic features are more consistent with Behçet’s Disease phenotype (14). Unfortunately, no skin biopsy results were obtained in this patient, so there is no direct evidence of vasculitis. The intestinal mucosal biopsy indicated an inflammatory response predominantly involving lymphocyte infiltration, but no granulomas typical of CD were observed. According to the International Criteria for Behçet’s Disease 2014 (12), our patient met four points. However, according to CD diagnostic criteria, the patient only meets the criterion for discontinuous, segmental changes, without the cobblestone appearance or longitudinal ulcers, and there is no evidence of transmural inflammation. Non-caseating granulomas, fistulas, or perianal disease were not observed. Therefore, the patient was managed as BD. In the 2016 pediatric BD criteria, our patient met only two of the six items outlined. One potential limitation is that approximately 80% of the patients with BD used to formulate the criteria were from the Middle East, where gastrointestinal involvement is infrequent (15). Hence, it would be advantageous for new criteria to consider symptom prevalence and the geographical distribution of patients.

In pediatric patients with early onset and multi-system involvement, the possibility of monogenic Behçet’s disease and a board range of inborn errors of immunity should be strongly considered, including chronic granulomatous disease, A20 haploinsufficiency, NEMO deficiency, IL21 deficiency, IL10 deficiency, IL10R deficiency, ELF4 deficiency, NFAT5 haploinsufficiency, TGFB1 deficiency, and others (16–20). Whole exome sequencing and karyotype analysis were performed on this patient, but no variants associated with the phenotype were identified, including those in *TNFAIP3, ADA-2, MEFV, NOD2, OTULIN, CYBB, NEMO, NFKB1, RELA, WDR1, IKBKG, GLA*, *IL10, IL10RA, IL10RB, RIPK1, TGFB1, ELF4, NFAT5, IL21*, etc. Additionally, no structural variants, such as Trisomy 8, were observed. Due to the overlapping symptoms between Behçet’s disease and other autoinflammatory and immunodysregulatory disorders, this patient was described as a Behçet’s disease phenotype. With the advancement of gene sequencing technology, an increasing number of new monogenic genetic diseases are being identified (20, 21). Considering the limitations in sequencing depth and genetic technology, there remains a possibility that a genetic etiology may be overlooked in this case. Therefore, newly identified pathogenic genes associated with Behçet’s disease should be continuously monitored, and vigilance should be maintained for the possibility that an as-yet unrecognized form of inherited etiology may exist in this child.

In addition to the common BD symptoms of recurrent oral ulcer and gastrointestinal ulcer, the boy experienced multiple aseptic abscesses (AAs). AAs are characterized by deep, sterile, round lesions comprising neutrophil infiltration, which do not respond to antibiotic therapy but improve with corticosteroid and immunosuppressive drugs (22). They often manifest with fever, weight loss, and abdominal pain and have been described in inflammatory bowel disease, particularly in Crohn’s Disease, as well as in Sweet’s syndrome and pyoderma gangrenosum (23, 24). Diagnosing AAs relies on exclusion criteria, including: (1) deep abscess evident on radiologic examination, with neutrophilic features confirmed by surgical pathology or aspiration when performed; (2) negative cultures and serologic tests; (3) failure of antibiotic therapy; (4) rapid improvement with corticosteroids (25). This patient met these criteria. Previous studies have reported several cases reported of AAs associated with BD (22, 23, 26). Besides multiple splenic abscesses, this boy also suffered from a rare gastric wall abscess, marking the first documented case of sterile gastric wall abscess associated with BD.

In patients with BD, parenchymal lung abnormalities are rare and may include atelectasis as well as nodular or reticular opacities (1). Aneurysms of the pulmonary arteries, either with or without thrombosis, are a typical manifestation of BD (27). In this patient, diffused ground-glass opacity was observed without apparent vascular lesions, diagnosed as ILD, which could potentially serve as an early sign of pulmonary involvement. Although parenchymal lung abnormalities have been reported in several cases of BD (27), such occurrences have not been documented in children until now.

Individualized multidisciplinary treatment is essential for managing this heterogeneous disease (28). Depending on the severity of the clinical manifestations and the presence or absence of factors associated with a poor prognosis, various strategies can be employed, including topical anti-inflammatory therapy, colchicine, glucocorticoids, and synthetic or biologic immunosuppressive agents (1, 6, 28). Our patient showed impressive improvement with prednisone treatment, not only for the multiple abscess but also for interstitial lung disease. There is no globally accepted therapeutic regime for the treating AAs in BD, but the literature strongly supports the use of glucocorticoids, which typically lead to rapid clinical resolution of symptoms followed by slower radiological improvement. Surgical intervention, including splenectomy, is also an option for refractory cases of aseptic splenic abscesses (22, 26, 29, 30). In cases of BD with pulmonary involvement, immunosuppressants and high-dose steroids have been reported to improve outcomes in a series of cases (27). Since ILD is considered to result from abnormal lung wound healing due to alveolar epithelial injury, and angiogenesis is involved in the pathogenesis of ILD (31), we believe that in our case the sustained remission of ILD after steroid discontinuation may be related to thalidomide maintenance therapy. However, the use of thalidomide in ILD currently lacks reliable evidence from clinical trials (32), which highlighting the need for future large-scale clinical studies to confirm this. Further robust clinical evidence is also needed to establish guidelines for managing pulmonary involvement (27).

## Conclusion

The presence of multiple AAs and pulmonary involvement may be the arising signs of a BD phenotype in childhood, and prompt use of glucocorticoids is effective in achieving a rapid clinical resolution. Long-term multidisciplinary monitoring is essential for differential diagnostic process with autoinflammatory and immunodysregulatory disorders in pediatric patients with early onset and multi-system involvement of the BD phenotype.

## Data Availability

The raw data supporting the conclusions of this article will be made available by the authors, without undue reservation.
